# Evaluating carboplatin and PARP inhibitor combination efficacy using high-grade serous carcinoma spheroids and organoids

**DOI:** 10.1080/15384047.2025.2611602

**Published:** 2026-01-11

**Authors:** Emily J. Tomas, Jennifer Davis, Yudith Ramos Valdes, Trevor G. Shepherd

**Affiliations:** a Cancer Research Laboratory Program, the Mary and John Knight Translational Ovarian Cancer Research Unit, Verspeeten Family Cancer Centre, London, Canada; b Department of Anatomy & Cell Biology, Western University, London, Canada; c Department of Oncology, Western University, London, Canada; d Department of Obstetrics & Gynaecology, Western University, London, Canada

**Keywords:** PARP inhibitors, high grade serous carcinoma, epithelial ovarian cancer, spheroids, organoids

## Abstract

**Background:**

PARP inhibitors (PARPis) are new targeted agents that exploit homologous recombination DNA repair deficiencies (HRDs), which are present in 50% of high-grade serous carcinoma (HGSC) cases. Currently, olaparib is approved as maintenance therapy for *BRCA1/2*-mutated HGSC, and niraparib is approved for platinum-sensitive recurrent disease. However, research is currently expanding their potential as front-line agents or in combination with carboplatin, a standard HGSC chemotherapeutic.

**Methods:**

Immortalized ovarian cancer (iOvCa) cell lines, developed from HGSC patient ascites, were treated with carboplatin, olaparib and niraparib to determine their sensitivity. Immunofluorescence analysis of RAD51 was conducted for HRD testing of all the cell lines. The cell lines were cultured as three-dimensional organoids and spheroids to mimic tumor growth and metastasis, respectively, and then treated to assess the effects of different drug combinations.

**Results:**

The half-maximal inhibitory concentrations of olaparib and niraparib varied across our iOvCa cell lines, with iOvCa195 *BRCA1*-mutant line exhibiting the expected high sensitivity to both PARPis. Direct combination of carboplatin with olaparib or niraparib enhanced cell killing, yet achieved cell viability levels to those of carboplatin alone. In sequential experiments, carboplatin followed by either PARPi or *vice versa* showed no significant difference in cell viability to carboplatin alone, except in iOvCa195 organoids when treated with a PARPi first.

**Conclusions:**

Overall, first-line carboplatin treatment remains ideal, yet there may be select utility for PARPi prior to chemotherapy. Using patient-derived tumor models such as spheroids and organoids may provide insights for on-going and future clinical trials to enhance therapeutic outcomes for HGSC patients.

## Introduction

Epithelial ovarian cancer (EOC) ranks among the deadliest cancers among women, with a survival rate less than 30%, largely due to late-stage detection.^1^ High-grade serous carcinoma (HGSC) is the most common subtype of EOC, representing 70%–80% of cases.^2^ This disease is characterized by universal *TP53* gene mutations and extensive chromosomal abnormalities. The standard treatment protocol for all EOC consists of surgical debulking with adjuvant chemotherapy combined with carboplatin and paclitaxel.^3^ This combination treatment is effective at targeting aggressive cancer cells, such as HGSC, because carboplatin is a platinum-based DNA alkylating agent and paclitaxel is an antimicrotubule agent, which together kill actively dividing cells. Even though this chemotherapy regimen can be successful for most patients initially, they often recur with platinum-resistant disease.^4^ The patients are then limited to second-line chemotherapeutics, including docetaxel, etoposide, gemcitabine or topotecan,^5^ yet these very rarely afford meaningful effects on survival. Thus, most research has been conducted to either discover novel targeted agents or determine new combinations of clinically approved drugs for HGSC.

Poly ADP-ribose polymerase inhibitors (PARPis) are targeted agents used for HGSC patients, typically in a maintenance setting. PARPis have been utilized for patients with homologous recombination DNA repair deficiency (HRD), which is usually hallmarked by a *BRCA1/2* mutation. Yet, HRD can be acquired through other mutations, such as those targeting *ATM, ATR, CHEK2, BRIP1, RAD51C,* and *PALB2* genes.^6^^,^^7^ Using PARPi in the context of HRD cancers exploits synthetic lethality, as they are unable to effectively repair both single-stranded and double-stranded DNA breaks, respectively. With deficient repair mechanisms for these breaks, cells accumulate DNA damage and induce cell death through apoptosis. Since approximately 50% of HGSC patients are classified as HRD-positive because of sporadic *BRCA1/2* mutations or HRD-associated mutations, PARPis have been approved as maintenance therapy for patients with these mutations. Olaparib was clinically approved for *BRCA*-mutant carriers with recurrent platinum-sensitive disease; in contrast, Niraparib was approved for all platinum-sensitive recurrent HGSC patients.^8^^,^^9^ Based on current clinical trials on HGSC, Niraparib has a higher affinity for trapping PARP1 on DNA than olaparib, but with increased adverse effects. Additionally, patients may develop resistance to PARPi where they become HR-proficient (HRP) through a variety of mechanisms.^10-12^ Hence, it may be useful to determine whether PARPi treatment prior to standard chemotherapy or in direct combination with carboplatin will benefit HGSC patients.

In recent years, patient-derived organoids (PDOs) have been harnessed for drug testing of many targeted agents, including PARPi.^13^ PDOs have a unique ability to recapitulate patient tumors *ex vivo* for a direct estimate of the response rate.^14-16^ A few studies have demonstrated that PARPi efficacy can be predicted, and potential resistance mechanisms (e.g., replication fork protection) can be elucidated rapidly using PDOs.^7^^,^^17^ Using a personalized medicine approach for HGSC, PDOs could be used in the clinical setting to test a variety of targeted agents for directing treatment decisions.^18-21^ In addition, PDOs have the potential to determine predictive biomarkers for PARPi sensitivity in HGSC patients. Previously, we assumed that only patients who have *BRCA1/2* or other HRD-associated mutations benefit from PARPi use, but there may be genetic or molecular markers beyond DNA repair processes. For example, genomic methylation signatures may predict a *BRCA* mutant-like phenotype that correlates with PARPi efficacy.^22^ As well, Sheta et al. determined that an epithelial‒mesenchymal transition phenotype, specifically low N-cadherin expression, may predict PARPi sensitivity, as this was observed using matched PDOs and HGSC tumors.^23^

In the present study, we utilized patient ascites-derived immortalized ovarian cancer (iOvCa) cell lines to determine carboplatin and PARPi sensitivity within 3D spheroid and organoid *in vitro* model systems. We first noted vast heterogeneity in carboplatin, olaparib and niraparib efficacy, which was expected with patient-derived HGSC cell lines. To evaluate new clinical approaches, we tested organoids and spheroids treated with carboplatin and either olaparib or niraparib in direct combination or in sequence. Overall, we failed to determine a combination or sequence that led to additive or synergistic efficacy beyond that of carboplatin alone. However, in one ascites-derived cell line, iOvCa195, PARPi treatment followed by carboplatin showed increased efficacy, indicating the use of PARPis in a front-line setting. Our results indicate that PARPi may be used prior to standard chemotherapy as an alternative yet effective treatment regimen for select HGSC patients.

## Materials and methods

### Cell lines

**Figure 1.**
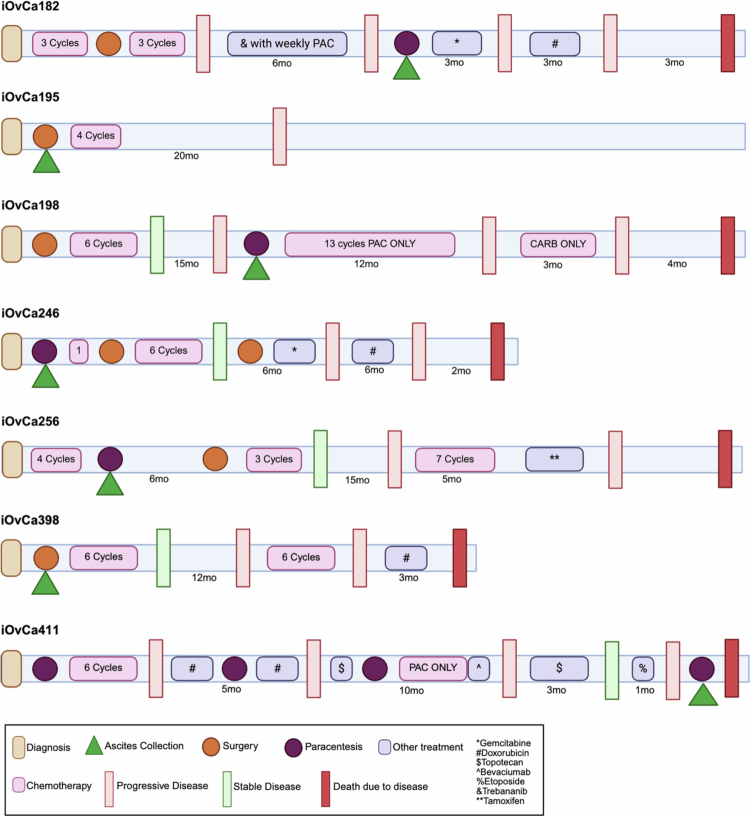
Clinical treatment course and timeline of HGSC patients. Each cell line was originally derived from the ascites samples of patients where clinical information was available and obtained with consent for their treatment course.

The following HGSC patient ascites-derived immortalized cell lines were used: iOvCa182, iOvCa195, iOvCa198, iOvCa246, iOvCa256, iOvCa398 and iOvCa411.^24^ iOvCa cell lines were derived from malignant ascites fluid collected during debulking surgery and continuously passaged in T-75 flasks (Sarstedt, Newton, NC, USA) until they were immortalized.^25^ Patient consent for clinical specimens from which these cell lines were derived was obtained according to our institution research ethics board-approved protocol (#115904), and clinical information is provided in Table 1 and Figure 1. The cells were maintained at 37 °C and a CO_2_ concentration of 5% in adherent cultures on tissue culture-treated polystyrene plates (Sarstedt, Newton, NC, USA) with 10% fetal bovine serum (FBS) (Wisent, Saint-Jean-Baptiste, QC, Canada) in DMEM/F-12 (Gibco, Grand Island, NY, USA). All cell lines were validated by STR analysis (The Centre for Applied Genomics, The Hospital for Sick Children, Toronto, ON, Canada) and tested for mycoplasma routinely using the Universal Mycoplasma Detection Kit (ATCC; Cedarlane, Burlington, ON, Canada).

**Table 1.** Clinical characteristics and treatment regimen of high-grade serous carcinoma patients from whom iOvCa cell lines were derived.

Cell line	Histopathology (staging)	Treatment prior to collection	Adjuvant treatment 1	Adjuvant treatment 2	Adjuvant treatment 3	Current status
iOvCa182	HGSC (IIIB)	6 cycles of carboplatin & paclitaxel; trebananib, angiopoietin inhibitor + weekly paclitaxel for 6 months (TRINOVA1)	3 cycles of carboplatin & gemcitabine	3 cycles of doxorubicin		Deceased (due to ovarian cancer)
iOvCa195 *(BRCA1mut)*^a^	Mixed HGSC and endometrioid (IV)	None	4 cycles of carboplatin and weekly paclitaxel			Alive with unknown disease status
iOvCa198	HGSC (IIIB)	6 cycles of carboplatin & paclitaxel	Weekly 1/2 dose paclitaxel for 1 y	3 cycles of carboplatin		Deceased (due to ovarian cancer)
iOvCa246	HGSC (IIIC)	None	6 cycles of carboplatin & paclitaxel	3 cycles of carboplatin & gemcitabine	4 cycles of doxorubicin	Deceased (due to ovarian cancer)
iOvCa256	HGSC endometrial (IIIA)	4 cycles of carboplatin and weekly paclitaxel	3 cycles of carboplatin & paclitaxel	7 cycles of carboplatin & paclitaxel		Deceased (unknown cause)
iOvCa398	HGSC (IC)	None	6 cycles of carboplatin & paclitaxel	6 cycles of carboplatin & paclitaxel	3 cycles of doxorubicin	Deceased (unknown cause)
iOvCa411	HGSC (IIIC)	6 cycles of carboplatin & paclitaxel; 5 cycles doxorubicin; weekly taxol for 8 months; topotecan; etoposide				Deceased (due to ovarian cancer)

HGSC = high-grade serous carcinoma. The staging was based on the International Federation of Gynecology and Obstetrics (FIGO) staging system.

a The mutations in iOvCa cell lines were determined based on oncogene hotspot analysis. As previously described by Tomas et al.^24^

### Organoid culture

The cells were seeded and resuspended in Cultrex Basement Membrane Extract (BME) PathClear Type 2 (Cedarlane, Burlington, ON, Canada) as droplets. The droplets were overlaid with EOC organoid-specific media that contains advanced DMEM/F-12 (Invitrogen, Waltham, MA, USA) supplemented with B-27™ (Invitrogen, Waltham, MA, USA), forskolin (Cedarlane, Burlington, ON, Canada), GlutaMAX™ (Invitrogen, Waltham, MA, USA), HEPES (Wisent, Saint-Jean-Baptiste, QC, Canada), human EGF (Peprotech Inc., Cranbury, NJ, USA), human FGF-10 (Peprotech Inc., Cranbury, NJ, USA), nicotinamide (MilliporeSigma, Oakville, ON, Canada), N-acetyl-L-cysteine (MilliporeSigma, Oakville, ON, Canada), recombinant human Noggin (R&D Systems, Toronto, ON, Canada) and Y-27632 dihydrochloride (MilliporeSigma, Oakville, ON, Canada). The formulation for the EOC organoid-specific media has been published previously.^24^

### Adherent cell dose response curves

Carboplatin was provided by the London Health Sciences Centre (London, ON, Canada) and stored in a sterile saline stock solution at 4 °C. Olaparib (AZD2281) and niraparib (MK-4827) were purchased from Cedarlane (Burlington, ON, Canada). The stock solutions were separately prepared from powders dissolved in 100% dimethyl sulfoxide (DMSO), aliquoted, and stored at −80 °C for up to a maximum of six months. The cells were seeded at 2 × 10^3^–6 × 10^3^ cells/well on 96-well standard tissue culture plates (Sarstedt, Newton, NC, USA) and treated 24 h after plating. The cells were treated with a 3-fold serial dilution of carboplatin (0.01–1000 µM) for 3 d or olaparib/niraparib (0.01–1000 µM) for 7 d. An alamarBlue™ assay (Thermo Fisher Scientific, Mississauga, ON, Canada) with a 4-h incubation period, according to the manufacturer's recommendations, was completed for cell viability, and the raw data were normalized to the lowest drug concentration used as the control.

### Spheroid drug treatment experiments

Cells were seeded at 2.5 × 10^3^ cells/well in a 96-well round bottom Ultra-low Attachment® (ULA) with DMEM/F12+10% FBS. After 3 d, the spheroids were treated with their designed carboplatin IC_50_ for 3 d or with their olaparib/niraparib IC_50_ for 7 d. An alamarBlue™ assay (Thermo Fisher Scientific, Mississauga, ON, Canada) with a 24-h incubation period, according to the manufacturer's recommendations, was completed for cell viability, and the raw data were normalized to media or DMSO control.

### Organoid drug treatment experiments

The cells were seeded at 2.5 × 10^3^ cells/well in 10 µL of Cultrex® BME on 96-well standard tissue culture plates with EOC organoid-specific media. After 7 d, the organoids were treated with their designated carboplatin IC_50_ for 3 d or olaparib/niraparib for 7 d. An alamarBlue™ assay (Thermo Fisher Scientific, Mississauga, ON, Canada) with a 24-h incubation period, according to the manufacturer's recommendations, was completed for cell viability, and raw data were normalized to media or DMSO control.

### PARPi combination treatment

The cells were seeded at 2.5 × 10^3^ cells/well in 96-well round bottom ULA plates with DMEM/F12 + 10% FBS for 3 d spheroids or in 10 µL of Cultrex® BME on 96-well standard tissue culture plates (Sarstedt, Newton, NC, USA) with EOC organoid-specific media for 7 d as organoids. After the designated timepoints, the cells were treated with olaparib or niraparib at their 1 C_50_ values for 4 d and then with carboplatin at their IC_50_ values for another 3 d generating a total treatment period of 7 d. An alamarBlue™ assay (Thermo Fisher Scientific, Mississauga, ON, Canada) with a 24-h incubation period, according to the manufacturer's recommendations, was completed for cell viability, and the raw data were normalized to the media control. Brightfield images were taken at the endpoint using the Incucyte S3® System (Sartorious, Oakville, ON, Canada).

### PARPi sequential treatment

The cells were seeded at 2.5 × 10^3^ cells/well in 10 µL of Cultrex® BME on 96-well standard tissue culture plates (Sarstedt, Newton, NC, USA) with EOC organoid-specific media for 7 d as organoids. Then, the cells were treated with either a PARPi first (olaparib or niraparib) then carboplatin or carboplatin followed by a PARPi for a total treatment period of 10 d. The cells were washed with the previous drug in EOC organoid-specific media prior to adding the second drug. An alamarBlue™ assay (Thermo Fisher Scientific, Mississauga, ON, Canada) with a 24-h incubation period, according to the manufacturer's recommendations, was completed for cell viability, and the raw data were normalized to the media control. Brightfield images were taken at the endpoint using the Incucyte S3® System (Sartorious, Oakville, ON, Canada).

### Antibodies

RAD51 clone 14B4 (MA1-23271: 1:500) was purchased from Thermo Fisher Scientific (Mississauga, ON, Canada). Phospho-histone H2A.X at serine 139 clone JBW301 (05-636, 1:500) was purchased from MilliporeSigma (Oakville, ON, Canada). Geminin (52508, 1:800) was purchased from Cell Signaling Technology (Whitby, ON, Canada). The fluorophore-conjugated secondary antibodies, anti-rabbit IgG AlexaFluor™ Plus 488 (A32731) and anti-mouse IgG AlexaFluor™ Plus 647 (A32728) were purchased from Thermo Fisher Scientific (Mississauga, ON, Canada).

### Immunofluorescence

Sterile 1.5 mm square glass coverslips (VWR, 48366-227, Mississauga, ON, Canada) were placed on the bottom of each well of a 6-well plate (Sarstedt, Newton, NC, USA), and then the cells were plated at ~2 × 10^5^ cells/well. The cells were treated with either 5 Gy of radiation with a 2-h resting period or treated with DMSO or 5 µM olaparib for 24 h. The cells were washed with Hank's balanced salt solution (Wisent, Saint-Jean-Baptiste, QC), fixed using 4% neutral buffered formalin (Thermo Fisher Scientific, Mississauga, ON, Canada), permeabilized with 0.1% Triton X-100 in 1x PBS and blocked with 10% normal goat serum (Thermo Fisher Scientific, Mississauga, ON, Canada) for 1 h. Primary antibodies were diluted in antibody diluent (1% BSA in 0.1% Triton X-100 in 1× PBS) with a 1:1000 dilution of 4ʹ,6-diamidino-2-phenylindole (DAPI) (Millipore Sigma, Oakville, ON, Canada) and incubated overnight at 4 °C. The secondary antibodies were diluted in antibody diluent at 1:500 and incubated for 2 h at room temperature. Coverslips were mounted using ProLong Glass (Thermo Fisher Scientific, Mississauga, ON, Canada) on microscope slides. Fluorescent images were captured using a Leica DMI4000B inverted microscope (Teaneck, NJ, USA).

### Quantification of RAD51 foci

Nine fields of view were obtained from immunofluorescent (IF)-stained slides per cell line per treatment condition. A Cell Profiler (Broad Institute of MIT and Harvard, Boston, MA, USA) was used to identify nuclei based on DAPI staining, proliferating nuclei based on Geminin staining (SNuclei) and distinct RAD51 foci per Geminin-positive (GEM+) nuclei.^26^ The workflow is illustrated in Figure S1A. HRP was defined as >50% of the cell population was GEM+ and had >5 RAD51 foci per nucleus. HRD was defined as <20% of the cell population was GEM+ and had >5 RAD51 foci per nucleus. Intermediate HR status was defined as 20%–50% of the cell population to be GEM+ and had >5 RAD51 foci per nucleus. This classification was based on other published reports.^7^^,^^27^^,^^28^

### Synergy matrix

The cells were seeded as spheroids at 2.5 × 10^3^ cells/well in 96-well round bottom ULA plates with DMEM/F12 + 10% FBS for 3 d. The spheroids were treated with olaparib (0–75 μM) for 4 d and then with carboplatin (0–600 μM) for another 3 d, generating a total treatment period of 7 d. An alamarBlue™ assay (Thermo Fisher Scientific, Mississauga, ON, Canada) with a 24-h incubation period, according to the manufacturer's recommendations, was completed for cell viability, and the raw data were normalized to the media control. SynergyFinder 3.0 was used to calculate the overall synergy score for these two agents based on the individual combinations.^29^

### Statistical analysis

Graphs were generated using GraphPad Prism 10 (GraphPad Software, San Diego, CA, USA), and most data are expressed as the mean ± SEM from at least three independent experiments. Student's *t*-test, an ordinary one-way analysis of variance (ANOVA) or two-way ANOVA with Tukey's multiple comparisons test was performed, with results being considered significant at *p* < 0.05. Specific details on the graphs, statistical tests and significance values are provided in each figure legend.

## Results

### Heterogeneous drug sensitivity among HGSC cell lines

**Figure 2.**
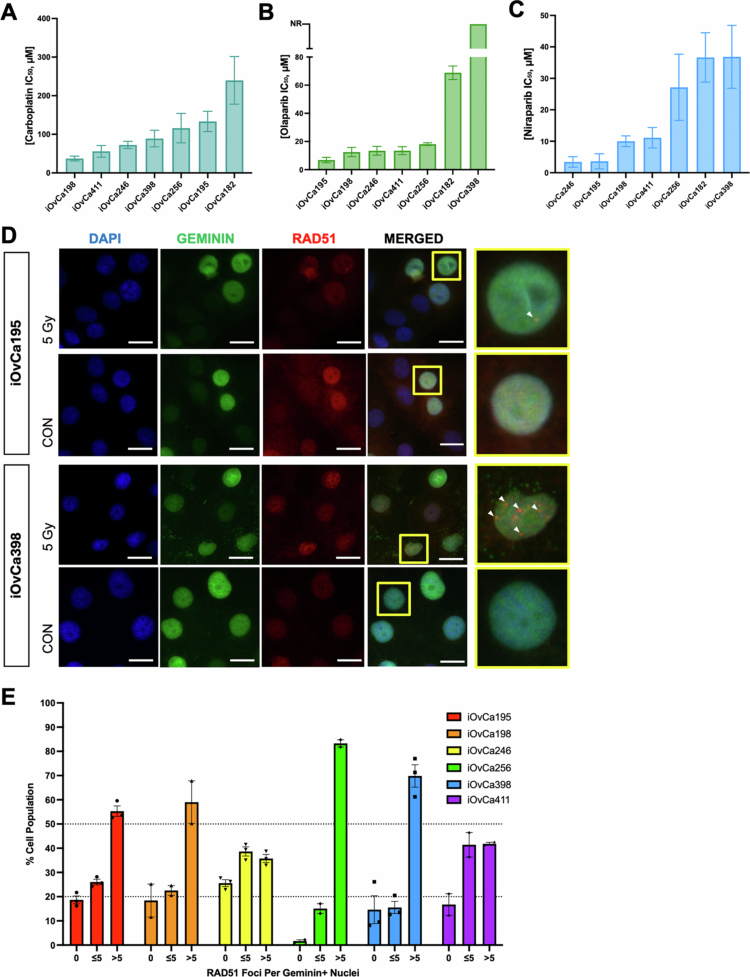
Sensitivity of carboplatin, olaparib, and niraparib on HGSC cell lines. Bar graphs of IC_50_ values for (A) carboplatin, (B) olaparib, and (C) niraparib in adherent culture conditions for each cell line based on alamarBlue readings from dose–response curves. (D) Immunofluorescence images of iOvCa195 and iOvCa398 cells after irradiation treatment with a 5 Gy dose demonstrating DAPI (blue), Geminin (green), and RAD51 foci (red). Yellow boxes indicate zoomed-in portions of the image, and white arrowheads indicate individual RAD51 foci. The scale bar is 20 μm. (E) Bar graph demonstrates the portion of the cell population that had zero (0), less than or equal to 5 (<5) or greater than 5 (>5) RAD51 foci per Geminin + nucleus. Each dot represents one slide. Graphs were generated using GraphPad Prism 10 demonstrating mean ± SEM for A, B and C (*n* = 3–4) or the mean ± SD for E (*n* = 2–3).

To determine differences in therapeutic efficacy, a panel of HGSC patient ascites-derived immortalized ovarian cancer (iOvCa) cell lines developed at our facility were treated with a range of carboplatin doses *in vitro*. It is well known that HGSC patients can respond well to chemotherapy treatment, especially carboplatin, which is often used as a maintenance therapy with recurrence.^30^ For platinum-resistant progressive disease, however, patients may receive several other treatments, as summarized in the clinical treatment courses relevant to the iOvCa cell lines used in this study (Table 1 and Figure 1). Since carboplatin is initially efficacious for most patients yet resistance is driven largely by this drug, most research into new drug combinations is done using carboplatin rather than paclitaxel.^31^ Thus, we sought to determine carboplatin dose‒response curves and IC_50_ value for each iOvCa cell line under adherent culture conditions. Representative curves are demonstrated for iOvCa195, iOvCa198 and iOvCa246 when treated with carboplatin (Figure S2A). Each cell line demonstrated unique sensitivity to carboplatin, with iOvCa198 being the most sensitive (IC_50_ = 37.24 μM) and iOvCa182 being the least sensitive (IC_50_ = 239.83 μM) (Figure 2A and Table S1). In fact, most cell lines had IC_50_ values, which would classify them as resistant to carboplatin (IC_50_ > 85 μM).^32^ As well, it was intriguing that therapy prior to ascites collection and cell line derivation did not correlate with carboplatin sensitivity. For example, the iOvCa182 and iOvCa411 cell lines were derived from patients who had undergone the most treatment courses prior to the collection date; iOvCa182 cells are quite resistant, yet iOvCa411 cells are relatively sensitive to carboplatin (Table 1 and S1).

Independently, we determined the sensitivity of these cell lines to two PARPi agents, olaparib and niraparib, which are approved for use in HGSC patients. Utilizing a similar protocol, dose‒response curves were generated to determine each cell line IC_50_ value for olaparib and niraparib, with representative curves for the same cell lines (i.e. iOvCa195, iOvCa198 and iOvCa246) (Figure S2B,C). Based on the PARPi mechanism of action, dose‒response curves were generated after a 7-d treatment period in comparison with a 3-d carboplatin treatment period.^13^^,^^33^ As expected, each cell line demonstrated its own unique sensitivity to a PARPi, and importantly, the only *BRCA1* mutant line, iOvCa195, was among the most sensitive cell lines to both olaparib and niraparib (Figure 2B,C). In contrast, iOvCa398 cells were either very resistant (IC_50_ > 25 μM) or nonresponsive (unable to calculate an IC_50_) to niraparib and olaparib, respectively.^34^ In general, we observed lower IC_50_ values in each cell line treated with niraparib as compared with olaparib, confirming that niraparib is a more potent PARPi. The only exception was iOvCa256 cells, which had a higher niraparib IC_50_ value of 27.16 μM in comparison with the olaparib IC_50_ value of 18.09 μM.

To determine if the inherent homologous recombination (HR) DNA repair status in our iOvCa cell lines correlated with PARPi sensitivity, immunofluorescent staining was conducted on irradiated adherent cells. Using a previously published protocol on a 5 Gy radiation treatment with a 2-h resting period, cells demonstrated an abundance of RAD51 foci in comparison to controls (Figure 2D).^35^ After quantification of the number of RAD51 foci per Geminin-positive (GEM+) nucleus, the mean number of RAD51 foci per GEM+ nucleus indicated that the more sensitive cell lines had less RAD51 foci (mean < 11), whereas the less sensitive lines had more RAD51 foci (mean > 11) (Figure S1B). Once sorted, the cell lines least sensitive to PARPi (i.e. iOvCa256 and iOvCa398) had >50% of the cells with >5 RAD51 foci per GEM+ nucleus, indicating that they are HRP (Figure 2E). Two cell lines had an intermediate HR status, with 20%–50% of cells having >5 RAD51 foci per GEM+ nucleus (i.e. iOvCa246 and iOvCa411). The remaining two cell lines (i.e. iOvCa195 and iOvCa198) were relatively sensitive to both PARPi based upon cell viability, yet the immunofluorescence results classify them as potentially HRP since they are at the threshold of >50% of cells with >5 RAD51 foci per GEM+ nucleus. This analysis was completed in conjunction with 5 μM olaparib treatment in comparison with the DMSO control to show increased RAD51 formation with a PARPi (Figure S1C). Additionally, phosphorylated-histone H2AX immunostaining demonstrated an increase in DNA damage under PARPi-treated conditions (Figure S1C). Overall, this analysis provides some insight into the HR status of cells and PARPi sensitivity.

### Spheroids and organoids have differing responses to carboplatin

**Figure 3.**
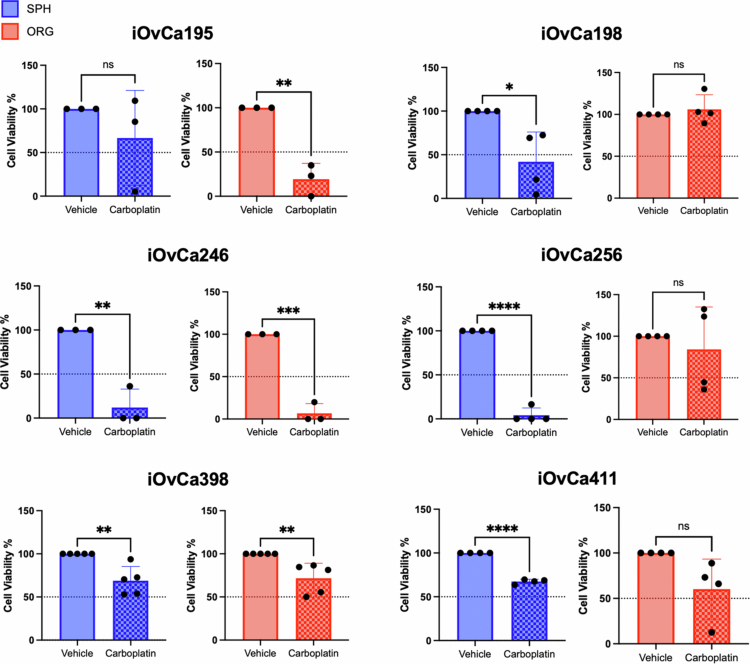
Carboplatin effects on HGSC cells cultured as spheroids or organoids. The cells were cultured as spheroids (blue) for 3 d or as organoids (red) for 7 d and then treated with the IC_50_ value as determined from adherent culture experiments for each cell line for an additional 3 d. The bar graphs show the change in cell viability normalized to that of the vehicle control. Graphs were generated using GraphPad Prism 10 to demonstrate the mean ± SEM with Student's t test for statistical analysis (*n* = 3-5, *p < 0.05, **p < 0.01, ***p < 0.001, and ****p < 0.0001).

Previous studies have shown that chemotherapy insult is a major stress stimulus for advanced EOC and that ascites-derived spheroids tend to be more resistant to these agents.^36^ In contrast, many EOC studies have demonstrated that *ex vivo* tumor-derived PDOs can accurately match patient response to treatment.^18^^,^^19^^,^^37^^,^^38^ Thus, we sought to evaluate any potential differences between these three-dimensional culture models in terms of disease-relevant therapeutic testing. iOvCa cells were cultured as spheroids and organoids to establish 3D structures and then treated for 3 d with carboplatin at their established adherent IC_50_ value. Interestingly, iOvCa195 spheroids appeared to be more resistant under this IC_50_ treatment condition (Figure 3). However, iOvCa198 and iOvCa256 cells showed the opposite results, with spheroids being more sensitive as compared with organoids. iOvCa398 and iOvCa411 cells cultured as spheroids and organoids demonstrated only moderate sensitivity to carboplatin, with >50% cell viability after treatment. Finally, iOvCa246 cells were very sensitive under both culture conditions, with almost no viable cells after carboplatin treatment. Representative images of iOvCa246 and iOvCa398 spheroids under treated and untreated conditions, provided in Supplementary Figure S3, demonstrates the changes in morphology that coincide with the differences in spheroid viability observed. Overall, the effects of carboplatin differ widely between these two 3D model systems.

### Carboplatin and PARPi has limited efficacy when combined

**Figure 4.**
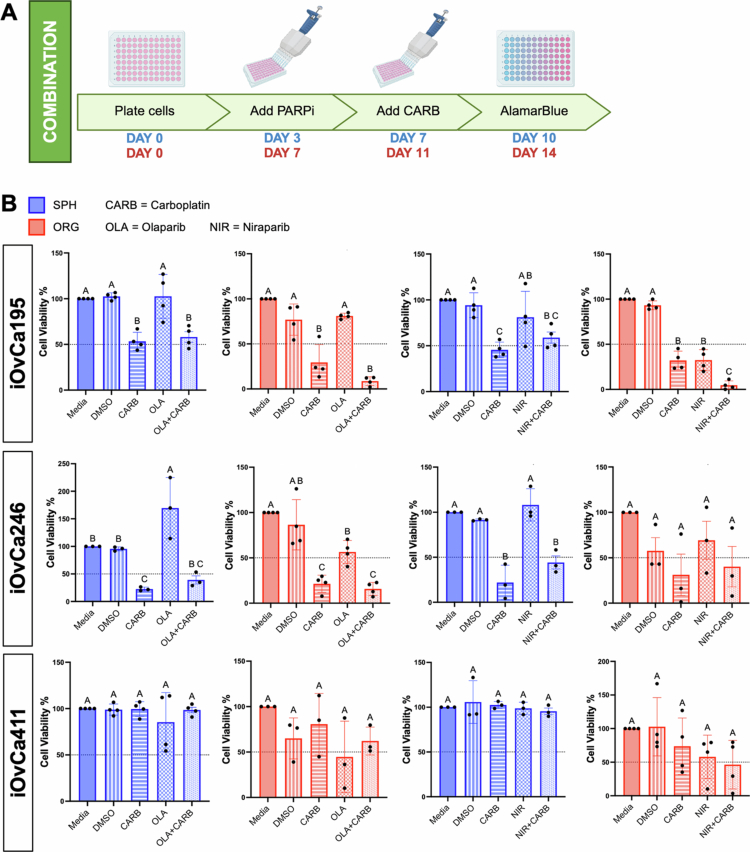
Direct combination of carboplatin with PARPi on HGSC spheroids or organoids. (A) The cells were plated as spheroids (blue) for 3 d or as organoids (red) for 7 d and then treated with the IC_50_ value (determined in adherent culture) of carboplatin (CARB) with either olaparib (OLA) or niraparib (NIR). For direct combination, they were treated with their PARPi IC_50_ value for 4 d and then with their carboplatin IC_50_ for the remaining 3 d. An alamarBlue assay was conducted at the treatment endpoint and read 24 h after addition. (B) Bar graphs showing the change in cell viability normalized to that of the media control. Graphs were generated using GraphPad Prism 10 demonstrate the mean ± SEM with an ordinary one-way ANOVA for statistical analysis and Tukey's multiple comparisons test displayed with compact lettering, where the same letters indicate nonsignificant differences and different letters indicate significant differences (*n* = 3–5).

In HGSC, carboplatin resistance can be acquired through a variety of mechanisms, such as the upregulation of drug efflux pumps and genetic mutations in apoptotic or DNA repair pathways.^39^^,^^40^ To combat carboplatin resistance and increase therapeutic efficacy in HGSC patients, we proposed that combining carboplatin resistance with a PARPi directly could be beneficial. To this end, we treated spheroids and organoids with a direct combination of carboplatin and either PARPi, olaparib or niraparib to determine whether this combination would elicit additive or synergistic effects. We developed a protocol similar to our previous experiments and recently published PDO drug screening methodologies, where both a PARPi and carboplatin were used at the same time on fully formed spheroids or organoids^13^^,^^18^^,^^20^^,^^41^ (Figure 4A). The iOvCa411 spheroids and organoids had no significant difference in viability among the treatment groups as compared with vehicle control (Figure 4B). The iOvCa246 spheroids and organoids had similar response on cell viability when treated with carboplatin alone or when combined with either PARPi. The remaining iOvCa cell lines (i.e. iOvCa182, iOvCa198, and iOvCa256) cultured only as organoids followed this same trend (Figure S4A–C). However, iOvCa195 organoids displayed increased efficacy with niraparib and carboplatin direct combination treatment; although not significantly different, there was an apparent trend for the same effect with olaparib and carboplatin in the iOvCa195 organoids (Figure 4B). This finding indicates that a direct combination of carboplatin and a PARPi may not be effective for most HGSC tumors, but only in specific cases.

To further interrogate carboplatin in direct combination with a PARPi, we conducted a dose–response matrix to determine whether these drugs may act synergistically to cause cell death but at alternative drug concentration ratios. We tested iOvCa195 spheroids in a direct combination with carboplatin and olaparib, since we observed equivocal results for spheroids, yet some evidence of an effect in organoids (Figure 4B). Based on the calculated Loewe synergy score (mean = −5.88; *p* = 3.23e-03) and ZIP synergy score (mean = −5.76; *p* = 1.84e-04), carboplatin and olaparib may be moderately antagonistic, especially when concentrations close to the iOvCa195 adherent IC_50_ values for each agent are used (Figure S5A,B). This finding indicates that carboplatin and olaparib are ineffective when used together, particularly in the context of dormant HGSC spheroids.

### Evaluation of carboplatin and PARPi sequential treatments

**Figure 5.**
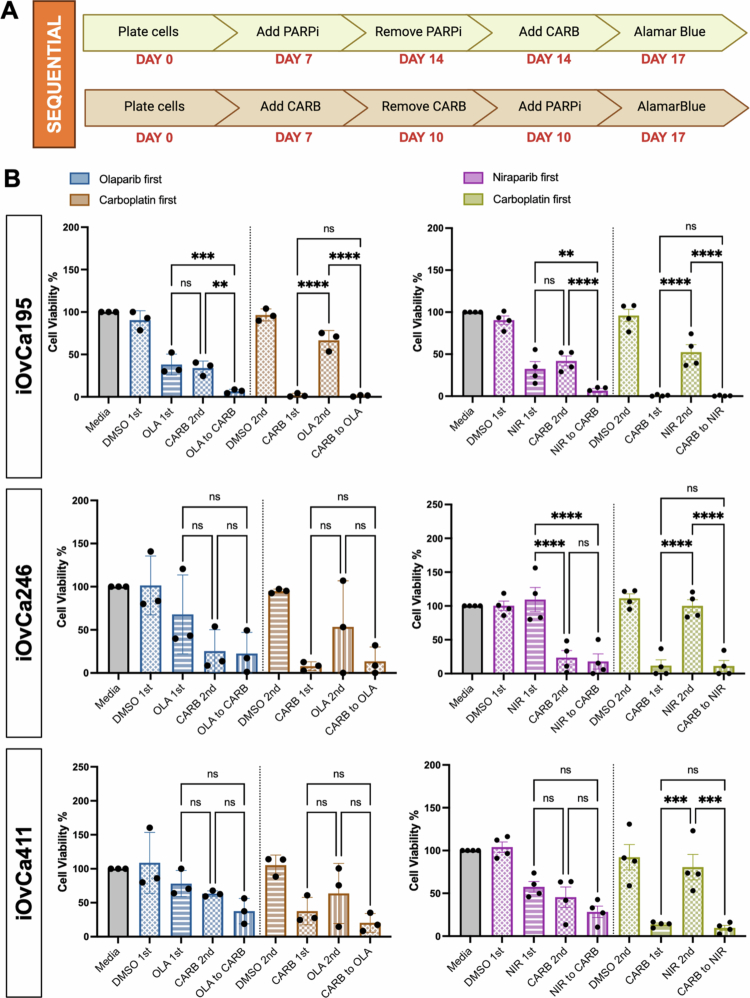
Sequential treatment of carboplatin with PARPi on HGSC organoids. (A) The cells were cultured as organoids for 7 d and then treated with the IC_50_ value (determined in adherent culture) for carboplatin (CARB) in sequence with either olaparib (OLA) or niraparib (NIR). For a sequential treatment, the cells were only treated either with carboplatin followed by a PARPi or with a PARPi followed by carboplatin as indicated for a 10-d total treatment period. An alamarBlue assay was conducted at the treatment endpoint and read 24 h after addition. (B) Bar graphs showing the change in cell viability normalized to that of the media control. Graphs were generated using GraphPad Prism 10 demonstrating mean ± SEM with a two-way ANOVA for statistical analysis (*n* = 3-4, *p < 0.05, **p < 0.01, ***p < 0.001, and ****p < 0.0001).

Our results indicate that the direct use of carboplatin and PARPi together may not be an effective treatment for HGSC. However, recent clinical trials have started to test up-front PARPi use prior to the initiation of standard chemotherapy rather than only maintenance therapy following chemotherapy.^9^ To address this new approach, we adjusted our protocol to reflect a sequential treatment with either carboplatin before PARPi or PARPi prior to carboplatin (Figure 5A). We opted to test this sequential treatment in organoids only, as both 3D models showed relatively similar effects in our hands, and we sought to align with PDO preclinical models for personalized medicine decisions. To visualize the effects of drug treatment on the organoids, brightfield images were captured, which showed differences in organoid size between the first treatment group and the sequential combination group (Figure S6). Notably, organoids of all three iOvCa cell lines demonstrated that carboplatin treatment prior to niraparib achieved the same cell killing effect as carboplatin alone (Figure 5B). Although iOvCa246 and iOvCa411 displayed no enhancement of organoid cell death due to the addition of either PARPi as compared with carboplatin alone, iOvCa195 organoids exhibited improved cell killing when treated with either olaparib or niraparib prior to carboplatin. This response aligned with our previous direct combination experiment where enhanced cell killing was observed with carboplatin and niraparib treatment in iOvCa195 organoids. As such, early PARPi use before carboplatin may be effective in very specific HGSC cases.

## Discussion

HGSC patients with advanced-stage disease undergo aggressive debulking surgery in combination with multiple rounds of cytotoxic chemotherapy using carboplatin and paclitaxel, which initially can be effective.^40^ However, the recurrence of chemoresistant disease is common, leaving patients with limited secondary treatment options. In this study, we evaluated carboplatin with a PARPi as a clinically relevant targeted agent to determine its efficacy as a combination treatment for HGSC and to test the efficacy of a PARPi prior to chemotherapy. We correlated PARPi sensitivity with HGSC patient ascites-derived cell line HR status through IF staining of RAD51 foci induced by gamma irradiation. Our results showed that HGSC lines were generally more sensitive to niraparib than to olaparib, which agrees with niraparib having a slightly better ability to trap PARP1 on DNA *in vitro.*^42^ However, our results demonstrated that the direct combination of PARPi and carboplatin was similar to carboplatin treatment alone. Shifting to a sequential treatment approach yielded similar results, but the use of a PARPi prior to carboplatin was effective in one of our patient samples (iOvCa195 organoids). This finding indicates that PARPi could be used prior to chemotherapy, but this would be useful in select HGSC cases. We conclude that if alternative combinations of these agents are to be assessed, *in vitro* organoids are an appropriate model system to test these therapeutic strategies. We note one limitation of this study is that single drug concentrations, determined empirically using standard adherent cell culture, were then used to perform all treatment combinations in 3D culture. This caveat certainly affected our ability to interpret the potential benefits of combining PARPi treatment in a sequential fashion with carboplatin. Thus, future studies would warrant careful consideration of reduced carboplatin concentrations to better interpret the potential additive and synergistic effects of clinically-meaningful drug combinations.

It is well established that HGSC patients can have quite varied responses to standard chemotherapeutics^18^^,^^43^^,^^44^ This concept holds true in the present study, which uses patient ascites-derived immortalized cell lines that were developed in our facility. Each iOvCa cell line displayed a range of cell killing sensitivities to carboplatin, olaparib and niraparib in adherent cultures. This variability was exacerbated when the cells were cultured as spheroids and organoids because it is known that drug sensitivities also differ between 2D and 3D culture systems. For example, the iOvCa411 cell line was relatively sensitive to all agents under classical adherent cell culture conditions even though the original patient had multiple courses of chemotherapeutic treatments prior to ascites collection. However, when cultured as spheroids or organoids, this sample was more resistant to carboplatin and PARPi as monotherapies and in combination. Compared with 2D cultures, 3D models may exhibit varied drug sensitivity due to microenvironmental gradients, limited drug penetration, and preserved heterogeneity that includes quiescent and stem-like populations.^3^^,^^23^^,^^30^^,^^45^ In addition, we have already provided evidence that these iOvCa cell lines differ when cultured as spheroids or organoids based on their transcriptome, pathway analysis, cell growth and 3D morphology.^24^ These differences highlight the improved physiological relevance of 3D systems and underscore their value in generating more clinically predictive insights into therapeutic efficacy.

Discovery of the PARP proteins, PARP-1/2, was foundational to understanding single-strand DNA damage repair though base excision repair and led to the development of PARPi as anticancer pharmaceuticals.^46^ Early *in vitro* and *in vivo* studies on PARPi use indicated their potential as potent chemosensitizers.^42^ Subsequent research focused on combination with DNA damaging agents, such as chemotherapy and radiotherapy. In our direct combination experiments, a PARPi was added to cells 4 d earlier than carboplatin was added to reduce the highly cytotoxic effects of the platinum-based agent and allow both drugs to equivalently act on these cells at the same time. Although, we understand that this protocol may not reflect the true concomitant therapy used in the clinic. Additionally, we implemented a sequential drug treatment experiment in which PARPi was added separately either before or after carboplatin. Herein, we demonstrated that PARPi may not act as a chemosensitizer when used in direct combination with carboplatin. Although, there is limited knowledge as to how these agents may act in cells within a 3D context as cultured spheroids and organoids. Our results indicated that PARPi and carboplatin had limited efficacy when used in combination in both spheroids and organoids, where most cell killing could be entirely due to carboplatin alone.

Our preliminary synergy matrix results using spheroid culture indicated that olaparib and carboplatin may possess moderately antagonistic effects. As such, a more thorough synergy matrix analysis would be beneficial to fully elucidate whether carboplatin may have potential synergism with either or both PARPis. Our analysis was limited by utilizing only the adherent IC_50_ values to determine the capacity of cells to respond to these agents when cultured as spheroids or organoids. However, it is well-known that specific drug combinations may yield synergistic effects, especially within 3D cultures where cells may be spatially regulated.^47^^,^^48^ This current study also provides the foundation for incorporating recently developed PDOs into any future high-throughput synergy analysis that could inform new clinical trials on PARPi.

The two approved PARPis used in HGSC are olaparib and niraparib, which vary in their selectivity and potency for trapping PARP1/2.^49^ Olaparib is recommended for patients with germline or somatic tumor mutations in *BRCA1/2*, whereas niraparib is recommended for patients with recurrent HGSC of any genetic status. More recently, the NOW clinical trial is actively investigating olaparib in the neoadjuvant setting.^50^ In addition, the NANT and OPAL clinical trials are investigating niraparib in sequence with carboplatin on confirmed HRD-positive patients with advanced disease.^51^ Therefore, we expanded our experiments to include sequential treatments to determine whether PARPi may still act as a chemosensitizer, yet in a different temporal context. In our study, we adopted a similar approach for our sequential experiments to determine the utilization of either of these two PARPi, especially since other *in vitro* studies on ovarian cancer have demonstrated synergy with pretreatment of a PARPi to platinum-based chemotherapy.^52^^,^^53^ Our results indicated that the use of a PARPi, either with olaparib or niraparib, prior to carboplatin was effective for one HGSC sample, the iOvCa195 organoid. This could potentially be explained by its *BRCA1* mutation or independent mechanisms such as PARP trapping toxicity, the latter of which could be evaluated in future mechanistic testing.^54^^,^^55^ Of course, the sample size would need to be greatly expanded to establish the potential of this treatment strategy in the clinical setting.

It is now considered that PARPi may be useful as a front-line maintenance therapy for those patients who are HRD-positive. PARP inhibitors exploit defects in HR repair through synthetic lethality, making HRD tumors particularly sensitive to treatment.^49^^,^^56^ *BRCA1/2* gene mutation is the primary marker used to establish PARPi sensitivity, with additional HRD testing representing a potential alternative. HR status assessment is therefore critical, as HRP tumors retain the capacity for DNA repair and generally show reduced responsiveness to PARP inhibition. Therefore, we implemented an immunofluorescence RAD51 foci functional HR assay to determine whether we could predict PARPi sensitivity. This assay was adopted from other reports that quantified RAD51 recruitment and foci formation to assess HR status in cells, where a large number of foci corresponds to HRP.^57^^,^^58^ Surprisingly, our analysis of radiation-induced RAD51 foci did not clearly correlate HR status with PARPi sensitivity in all cell lines. The HRD- and PARPi-sensitive lines were iOvCa246 and iOvCa411, in contrast to the HRP- and PARPi-resistant lines were iOvCa256 and iOvCa398. However, it did not show that iOvCa195 cells were HRD, even though they had a *BRCA1* mutation, indicating that HR-associated genes do not always correlate with HRD status. As well, RAD51 foci may not always align with PARPi sensitivity because the drug response can also be shaped by compensatory repair pathways, tumor heterogeneity, and the cell cycle context.^59-61^ These findings highlight the limitations of relying on a single HR biomarker and suggest that a multifactorial approach, including the mutational status of *BRCA1/2* and related DNA repair genes, may be necessary to accurately predict the PARPi response. Therefore, our results clearly show that this analysis would be complementary to mutational analysis, and more research needs to be conducted for additional biological predictors of PARPi sensitivity or resistance beyond HR DNA repair mechanisms.

PARPi resistance in HGSC arises through multiple mechanisms, including the restoration of HR function, increased drug efflux, replication stress adaptation, and tumor microenvironment changes.^4^^,^^17^^,^^62^ Some groups have determined that *BRCA* reversion mutations and loss of *BRCA1* promoter methylation can restore HR proficiency and reduce PARPi sensitivity.^22^^,^^63^ Additionally, the activation of ATR/CHK1 signaling and the stabilization of stalled replication forks via RAD51 upregulation contribute to resistance.^10^^,^^11^^,^^64^^,^^65^ This mechanism may explain our results using iOvCa398 cells as they were highly resistant to both olaparib and niraparib and showed a high density of radiation-induced RAD51 foci staining. Although, further verification would be required to assess replication fork stability *in vitro*. Hence, understanding these resistance pathways at the mechanistic level is crucial for optimizing PARPi efficacy and determining their appropriate use for specific HGSC tumors.

To expand and improve the efficacy of PARPi in the clinic, novel combinations with other agents may be needed. These include combination therapies with antiangiogenic agents, ATR inhibitors, immune checkpoint inhibitors, and HDAC inhibitors.^10^^,^^11^^,^^33^^,^^66-69^ For example, the ANITA/ENGO-OV41 phase III trial is investigating the novel application of the PD-L1 inhibitor atezolizumab to carboplatin-based chemotherapy followed by maintenance with niraparib to improve progression-free survival in patients with platinum-sensitive recurrent HGSC.^70^ In addition, the EFFORT study assessed the addition of olaparib to the Wee1 inhibitor adavosertib in PARPi-resistant high-grade serous ovarian cancer with some success and manageable adverse effects.^71^

## Supplementary Material

Supplementary Table 1.docx Supplementary Table 1.docx

Supplementary Figure Legends.docx Supplementary Figure Legends.docx

Supplementary material Supplementary Figure 3.tiff

Supplementary material Supplementary Figure 1.tiff

Supplementary material Supplementary Figure 6.tiff

Supplementary material Supplementary Figure 2.tiff

Supplementary material Supplementary Figure 4.tiff

Supplementary material Supplementary Figure 5.tiff.

## Data Availability

The authors confirm that the data supporting the findings of this study are available within the article and its supplementary materials. Any data that support this study are available from the corresponding author, TGS, upon reasonable request.

## Conclusions

In conclusion, our study demonstrated opportunities to test PARPi using alternative sequencing with chemotherapy for HGSC. Adjusting the standard treatment strategy may be necessary to improve progression-free survival for patients, but this is likely to be tailored to specific tumor biology. Further studies using patient-derived 3D culture models will uncover the mechanistic basis for PARPi combinations and identify useful predictive biomarkers to guide improved treatment decisions.
