# Supplementary material for: Evaluating carboplatin and PARP inhibitor combination efficacy using high-grade serous carcinoma spheroids and organoids
Source: Cancer Biol Ther. 2026 Jan 11;27(1):2611602. doi: 10.1080/15384047.2025.2611602 (PMC12795296; doi:10.1080/15384047.2025.2611602)
Supplement: Supplementary Table 1.docx [file KCBT_A_2611602_SM3019.docx]

**Supplementary Table 1. IC_50_ values for carboplatin, Olaparib and Niraparib in HGSC cell lines under adherent culture conditions.**

| **Cell Line** | **Carboplatin IC_50_ (μM)** | **Olaparib IC_50_ (μM)** | **Niraparib IC_50_ (μM)** |
| --- | --- | --- | --- |
| iOvCa182 | 239.73 | 68.81 | 36.64 |
| iOvCa195 * | 133.3 | 6.86 | 3.64 |
| iOvCa198 | 37.24 | 12.52 | 10.01 |
| iOvCa246 | 89.85 | 13.42 | 3.43 |
| iOvCa256 | 115.96 | 18.09 | 27.16 |
| iOvCa398 | 88.97 | NR | 36.84 |
| iOvCa411 | 55.87 | 13.49 | 11.13 |

* contains a germline *BRCA1*-mutation; NR = not responsive
