# Supplementary material for: Evaluating carboplatin and PARP inhibitor combination efficacy using high-grade serous carcinoma spheroids and organoids
Source: Cancer Biol Ther. 2026 Jan 11;27(1):2611602. doi: 10.1080/15384047.2025.2611602 (PMC12795296; doi:10.1080/15384047.2025.2611602)
Supplement: Supplementary Figure Legends.docx [file KCBT_A_2611602_SM3015.docx]

**Supplementary Figure Legends**

**Supplementary Figure 1. Quantification of RAD51 foci on immunofluorescently stained images.** (A) CellProfiler workflow demonstrating the software’s ability to (1) identify GEM+ nuclei (SNuclei), (2) identify RAD51 foci within each GEM+ nucleus and (3&4) mask then identify the RAD51 foci using per-object (SNuclei) thresholding and determining which foci belong to which nuclei for counting. (B) Violin plot demonstrating the range of the number of RAD51 foci per GEM+ nucleus in each cell line with the mean shown as a red line and the values provided above each plot. Graph was generated using GraphPad Prism 10. (C) Immunofluorescent images of iOvCa398 cells treated with 5μM of Olaparib or DMSO demonstrating DAPI (blue), Geminin (green), and RAD51 foci (red, left) or phosphorylated-histone H2AX (red, right). Scale bar is 20μm.

**Supplementary Figure 2. Dose response curves of three iOvCa cell lines treated with carboplatin, Olaparib, and Niraparib.** Dose response curves for (A) carboplatin, (B) Olaparib, and (C) Niraparib in adherent culture conditions for each cell line (i.e. iOvCa195, iOvCa198 and iOvCa246) based on alamarBlue readings normalized to lowest concentration of drug. Calculated IC_50_ values for each curve are indicated in each legend. Graphs were generated using GraphPad Prism 10 demonstrating mean±SEM with a sigmoidal interpolation analysis (n=3-4).

**Supplementary Figure 3. Brightfield images of iOvCa246 and iOvCa398 spheroids treated with carboplatin.** Spheroids were imaged using a Leica DMI4000B inverted microscope at the 3-day treatment endpoint. Scale bar is 200μm.

**Supplementary Figure 4. Direct combination of carboplatin and a PARPi on remaining HGSC organoids.** Cells were plated as organoids (red) for 7 days, then treated with the IC_50_ value as determined by adherent culture of carboplatin (CARB) with Olaparib (OLA). The cell line specific IC_50_ values for each drug are indicated underneath each cell line graph title. Bar graphs show the change in cell viability normalized to media control. Graphs were generated using GraphPad Prism 10 demonstrating mean±SEM with an ordinary one-way ANOVA for statistical analysis and Tukey’s multiple comparisons test displayed with compact lettering (n=3-4).

**Supplementary Figure 5. Dose response matrix for direct combination of carboplatin and Olaparib on iOvCa195 spheroids.** The synergy was calculated using the (A) Loewe and (B) ZIP models implemented in SynergyFinder3.0[29]. Green colour (<-10 synergy score) indicates antagonistic combination and red colour (>10 synergy score) indicates synergistic combination.

**Supplementary Figure 6. Brightfield images of iOvCa195 organoids under each sequential treatment group.** Organoids were imaged using our Incucyte® S3 System with the Organoid Software Module at the endpoint of the sequential treatment. Scale bar is 900μm.
