# Supplementary figures and images for: Evaluating carboplatin and PARP inhibitor combination efficacy using high-grade serous carcinoma spheroids and organoids
Source: Cancer Biol Ther. 2026 Jan 11;27(1):2611602. doi: 10.1080/15384047.2025.2611602 (PMC12795296; doi:10.1080/15384047.2025.2611602)

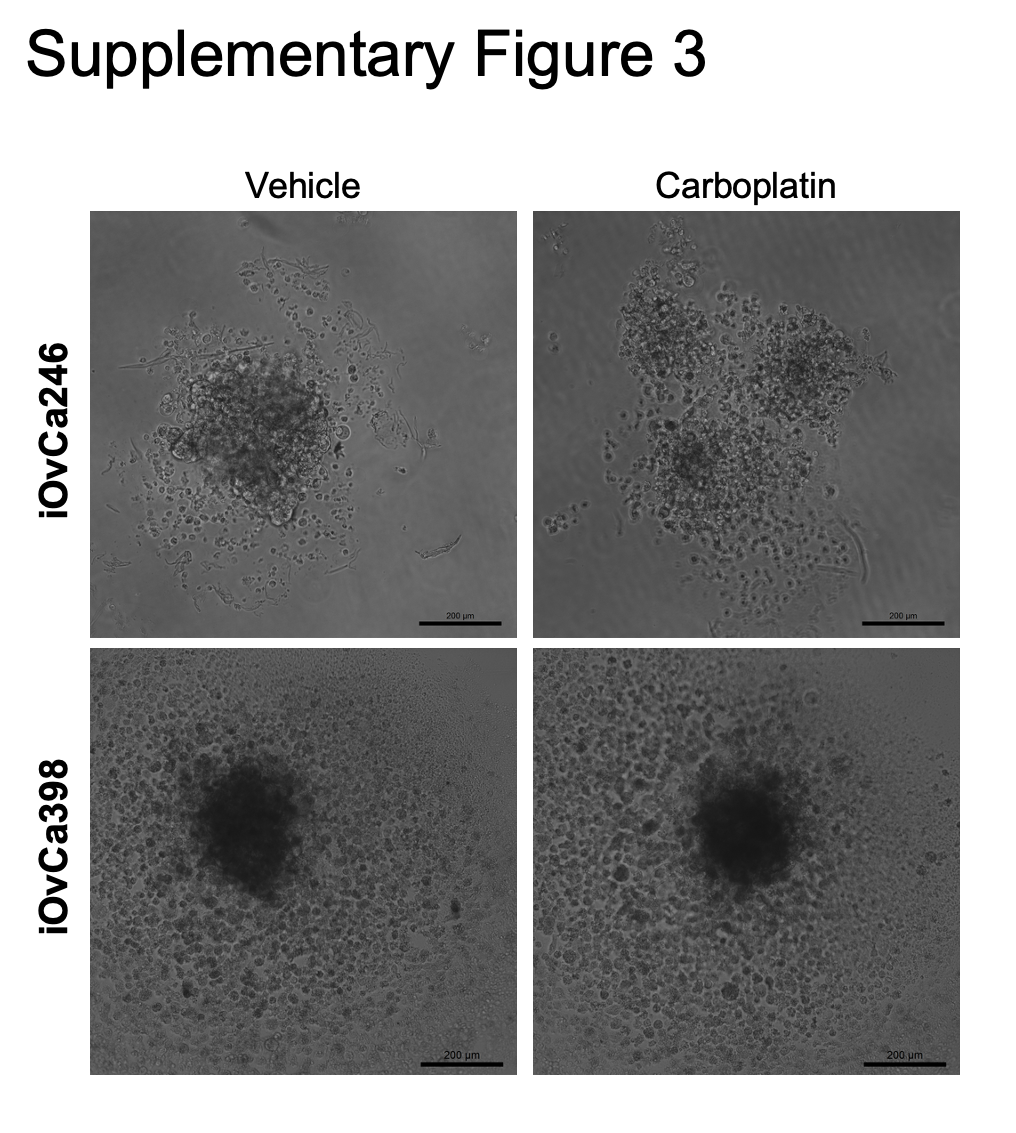

Supplement: Supplementary material — Supplementary Figure 3.tiff [file KCBT_A_2611602_SM3023.tif]

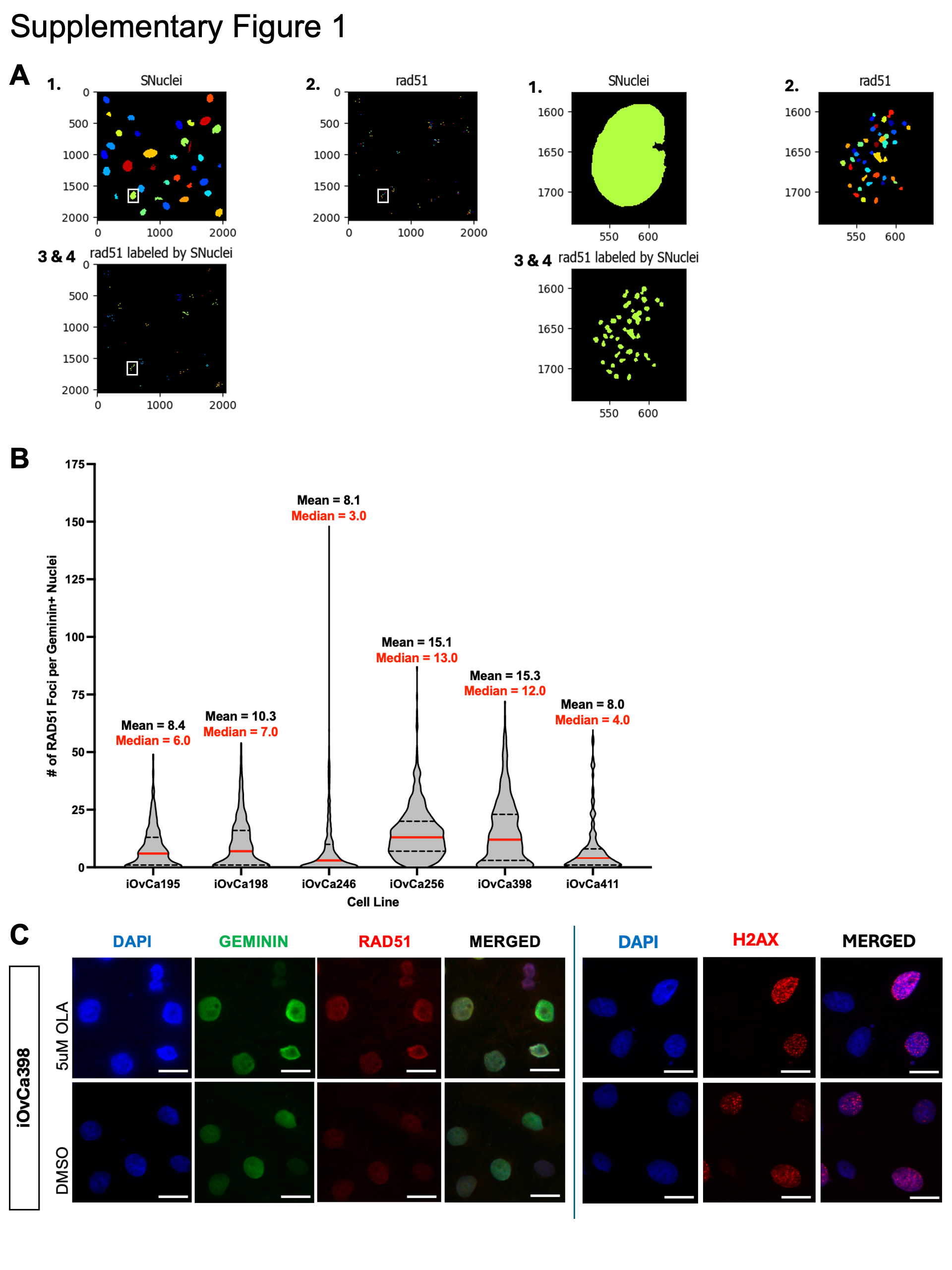

Supplement: Supplementary material — Supplementary Figure 1.tiff [file KCBT_A_2611602_SM3021.tif]

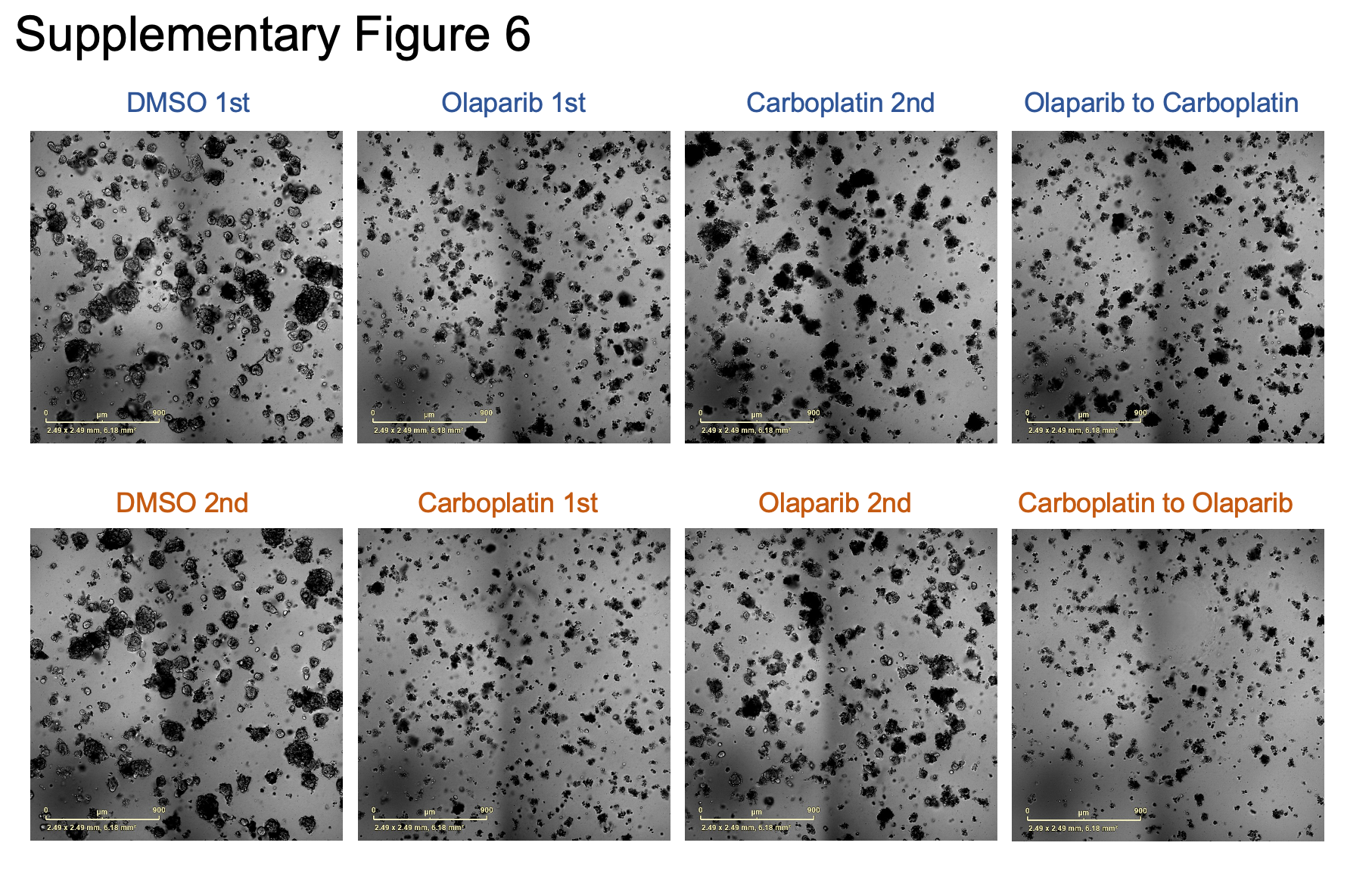

Supplement: Supplementary material — Supplementary Figure 6.tiff [file KCBT_A_2611602_SM3020.tif]

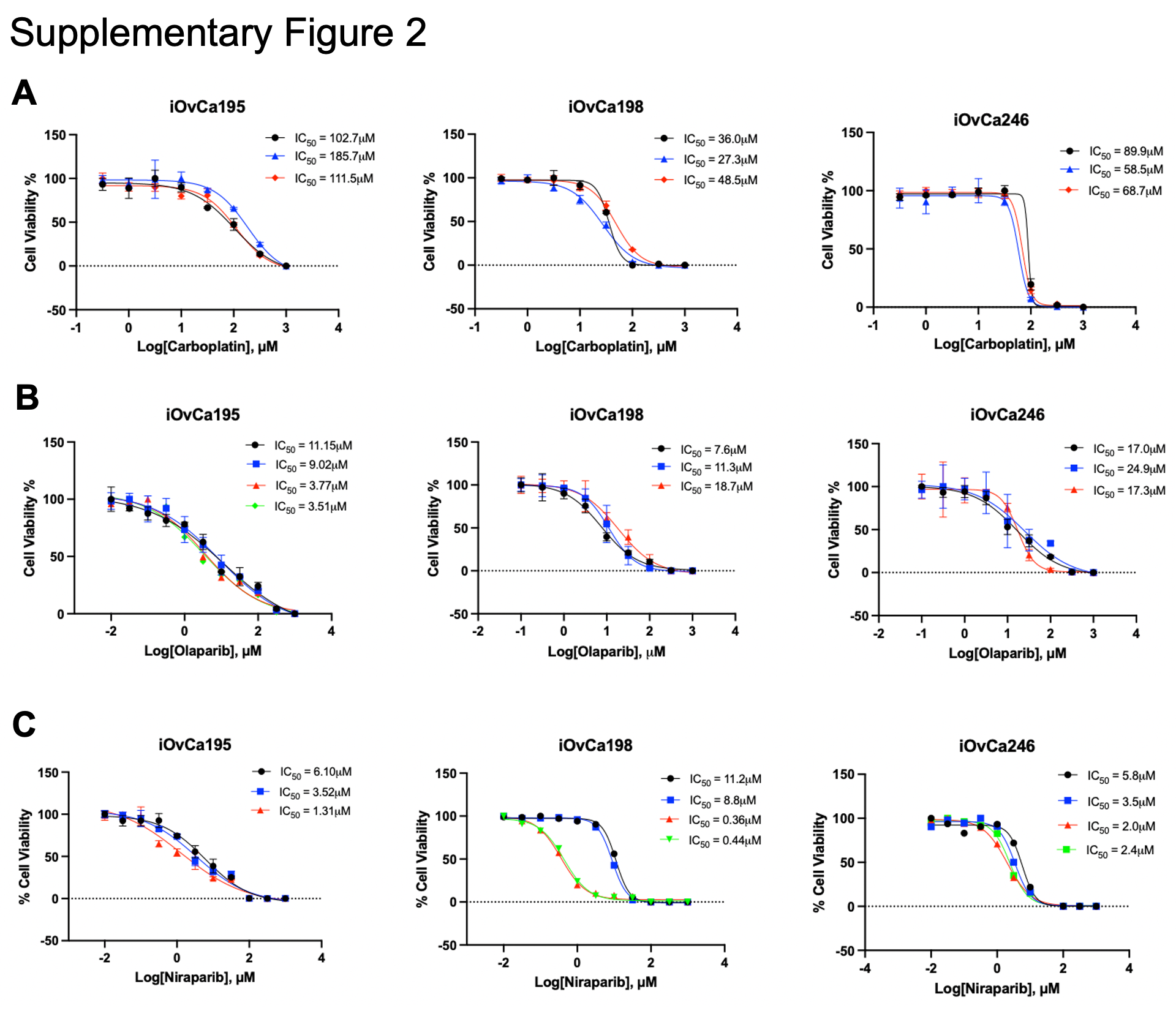

Supplement: Supplementary material — Supplementary Figure 2.tiff [file KCBT_A_2611602_SM3017.tif]

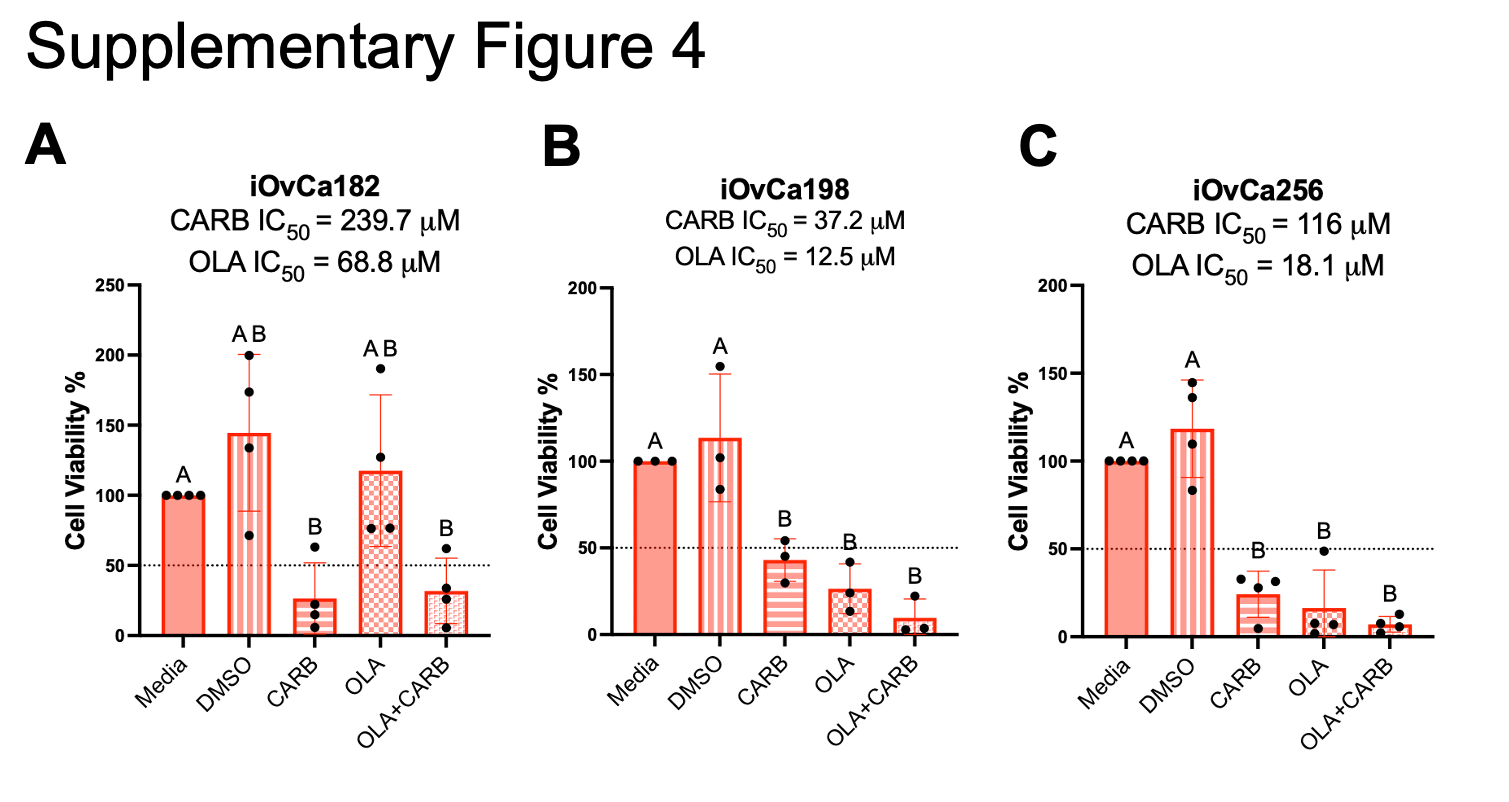

Supplement: Supplementary material — Supplementary Figure 4.tiff [file KCBT_A_2611602_SM3022.tif]

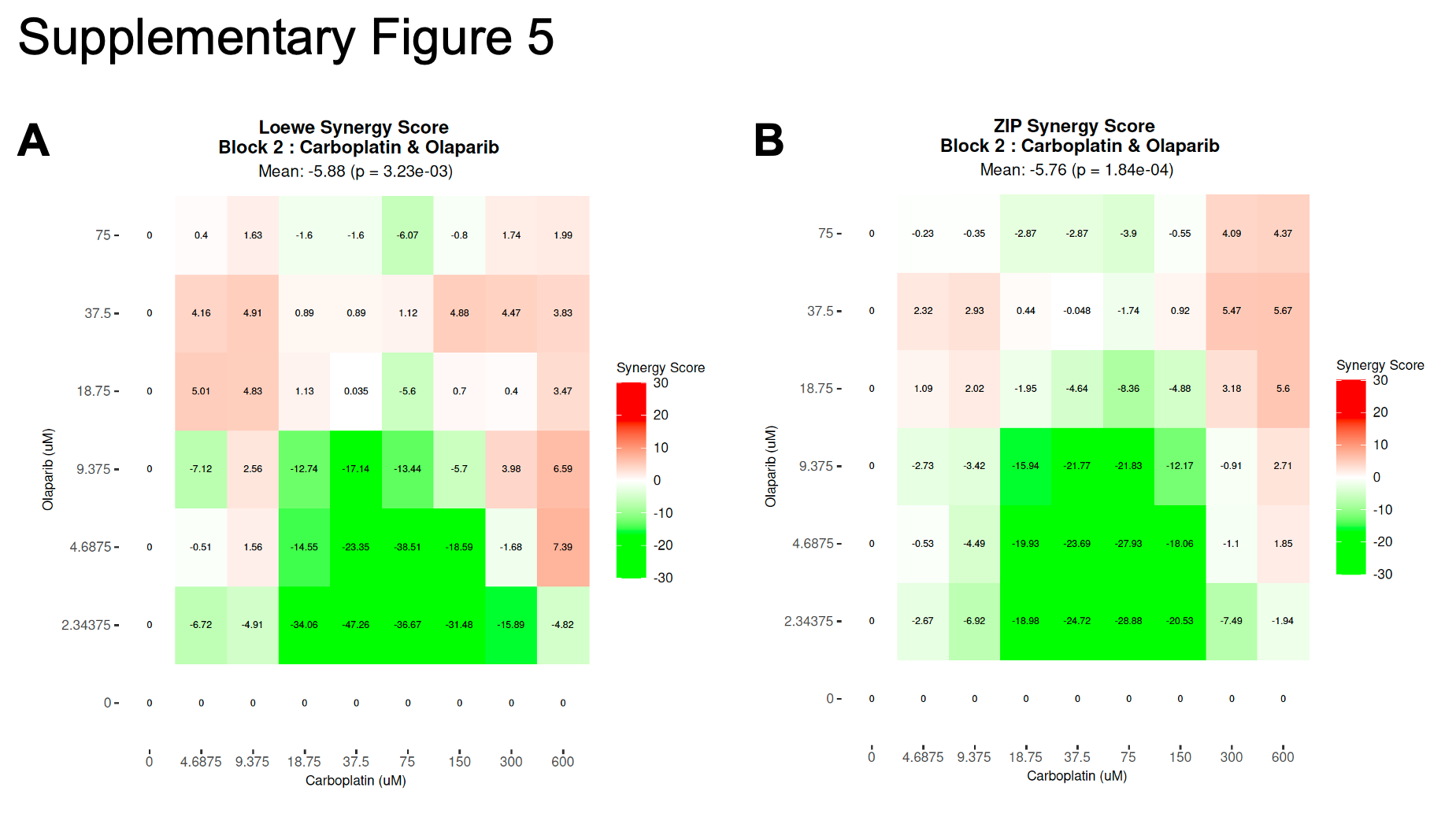

Supplement: Supplementary material — Supplementary Figure 5.tiff. [file KCBT_A_2611602_SM3016.tif]
